# Development and Usability of MSafe: A Fall Risk Application for Older Adults with Multiple Sclerosis

**DOI:** 10.3390/s25227075

**Published:** 2025-11-20

**Authors:** Katherine L. Hsieh, Deborah Backus, T. Bradley Willingham, Jon Sanford

**Affiliations:** 1 Department of Physical Therapy, College of Nursing and Health Professions, Georgia State University, Atlanta, GA 30303, USA; 2 Shepherd Center, Virginia C. Crawford Research Institute, Atlanta, GA 30309, USA; 3 Department of Occupational Therapy, College of Nursing and Health Professions, Georgia State University, Atlanta, GA 30303, USA

**Keywords:** smartphone, mobile health, aging, falls, neurodegenerative

## Abstract

**Background**: Falls are highly prevalent in older adults with Multiple Sclerosis (MS) and stem from a complex interplay of physiological, psychosocial, cognitive, and environmental risk factors. Fall risk assessments rely on in-person visits and occur infrequently, but mobile technology can provide portable, cost-effective, and multifactorial screening. The purpose of this study was to develop and evaluate the usability of a multifactorial fall risk app (MSafe) for older adults with MS. **Methods**: MSafe consists of 37 self-report questions, 9 quantitative cognitive and mobility assessments, and a final fall risk report. One-on-one semi-structured interviews were conducted with 21 older adults (>55) with MS. Participants independently used MSafe, were asked about their likes and dislikes, and completed the System Usability Scale (SUS). Interviews were video-recorded, transcribed, and coded into themes. **Results**: Three themes emerged: (1) simplicity of use, (2) progress monitoring, and (3) guidance and support. Overall, participants found MSafe easy to use, valuable to track and monitor their fall risk, and either confirmed or increased awareness of their own abilities. SUS scores averaged 84.9 ± 14.7. **Conclusions**: MSafe is a comprehensive fall risk app that demonstrated high usability by older adults with MS. Future steps include implementing MSafe in home settings to examine fall risk management.

## 1. Introduction

Multiple Sclerosis (MS) is a chronic autoimmune disease of the central nervous system [1]. While MS is most commonly diagnosed between the ages of 20 and 40, improvements in treatment and disease management have significantly altered the long-term prognosis for many living with MS [2]. As a result, individuals aged 55 years and older represent the largest, and fastest growing, portion of the MS population [3]. This demographic shift highlights an increasingly important, yet largely unaddressed, need for research and innovation targeting the needs of those aging with MS. Indeed, people with MS commonly experience a wide range of mobility, cognitive, vision, and sensory impairments that lead to reduced quality life while increasing the risk of secondary comorbidities and falls [1,4]. The physiological changes that come with natural aging often intensify the symptoms of MS and accelerate functional decline relative to younger individuals [5]. Meeting the needs of this growing population will require new approaches to care and research that are grounded in the realities of aging with a chronic neurological condition.

When considering the numerous factors associated with MS care in an aging population, the prevention of falls in older adults with MS has emerged as a key therapeutic target for promoting functional independence [6]. Across all ages, one in two people with MS experience a fall every 6-month period [7,8,9]. Over time, the occurrence of falls can lead to a chronic fear of falling, which in turn leads to activity curtailment and social isolation [10]. Falls often result in fractures, traumatic brain injury, and even death [6]. Due to these detrimental health consequences, preventing falls is critical to maintain functional independence [9].

A central challenge to managing fall risk in people with MS is that falls stem from a complex interplay of physiological, psychosocial, cognitive, and environmental risk factors that are difficult to quantify; therefore, identifying people with MS who may be at risk of falling and developing strategies to reduce the risk of falling in this population has remained a persistent and unresolved issue in MS care. While some strategies have been shown to successfully identify and mitigate fall risks in people with MS [11,12], current solutions depend heavily on in-person encounters, occur only at discrete time points, and provide limited ability to track the gradual and often subtle changes that influence fall risk in daily life. In addition, many people living with MS face obstacles to regular clinic attendance, and clinicians themselves frequently lack the time and resources needed for ongoing, detailed monitoring [13,14]. Collectively, these constraints leave current practices unable to deliver timely or individualized fall-risk detection and prevention for this population.

Advancements in mobile technologies, such as smartphone software applications (apps) and wearable sensors, offer new opportunities to expand clinical evaluation by delivering portable, cost efficient, and scalable solutions for older adults with MS to assess their fall risk [15,16]. Smartphone apps also have the ability to integrate data from built-in sensors such as accelerometers and gyroscopes to measure mobility. For instance, previous studies have leveraged smartphone sensors to assess postural control and dual-tasking abilities [15,17,18]. When coupled with self-reported inputs, apps have the potential to assess multiple fall risk domains. Moreover, smartphone and mobile health (mHealth) apps are frequently used by people with MS. Approximately 90% of people with MS own a smartphone [19], and about half use mHealth apps [20]. Given the accessibility and ability to continuously collect data during everyday activities, mobile apps have immense potential to overcome the limitations of contemporary in-clinic strategies and support more individualized, data-driven, and ecologically valid solution for fall management in people with MS.

**Figure 1:**
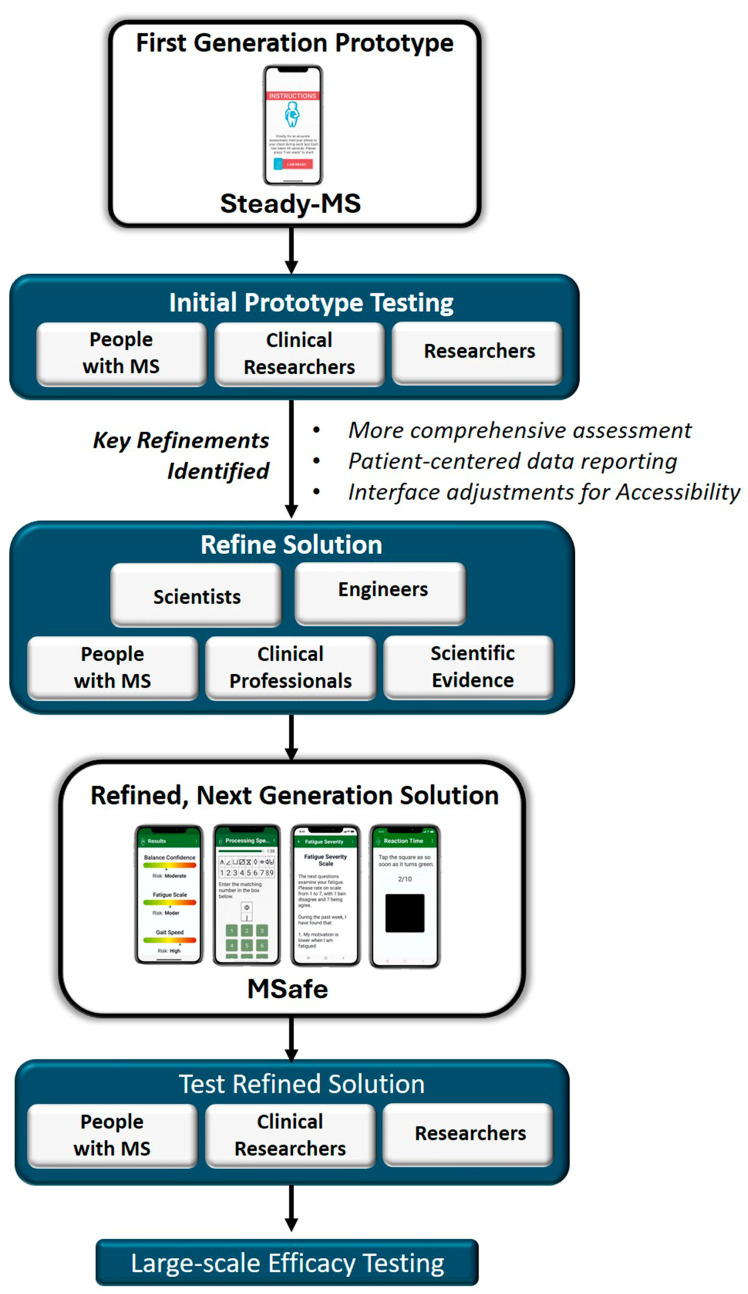
Steady-MS was first developed to assess postural control and self-reported questionnaires. Following initial prototype testing, we refined and developed MSafe with expertise from clinicians, engineers, and people with MS. We evaluated the usability of MSafe with older adults with MS, and future steps include large-scale efficacy testing in home settings.

In a prior study, we reported on the development of a fall risk app, Steady-MS^TM^, that assessed postural control during progressively challenging standing balance conditions and self-reported psychosocial outcomes [21]. While we found Steady-MS to be usable among young to middle aged adults with MS and valid compared to gold-standard force plates and inertial measurement units [21,22], Steady-MS measured a limited number of risk factors and was not tailored for the older MS population. Moreover, feedback from users and observations during testing highlighted several areas for improvement related to how information is presented back to users and design features that may enhance usability and accessibility. Moving forward, a major goal was to build a more complete self-check that includes both internal factors, like fatigue and attention, and external ones, including physical environmental factors, such as condition of walking surfaces, and presents results to users in a clear manner (Figure 1). Thus, the purpose of this study was to develop and evaluate the usability of a new iteration of our fall risk app (MSafe) for older adults with MS that includes the key features and considerations that were identified from our prior work. In addition to several practical adjustments to the user interface to accommodate the typical functional limitations associated with MS, this next-generation solution incorporates a comprehensive self-assessment to include both intrinsic and extrinsic fall risk factors (e.g., cognition, fatigue, environment) for more accurate fall risk assessments.

## 2. Materials and Methods

### 2.1. MSafe Development

MSafe was designed in Android Studio version Koala (2024.1.1 Patch 2, Google, Mountain View, CA, USA) and was written in Kotlin. MSafe leverages features of Android including the Sensor APIs (for accelerometer, gyroscope, and high sampling rate sensors) and the IIR Filter library (iirj 1.1) for advanced signal processing. The sampling rate for the accelerometer was 200 Hz. Two levels of filtering were used. A 4th-order Butterworth low-pass IIR filter with cut off frequency of 5 Hz was used, followed by a complimentary filter, fusing the gyroscope and accelerometer data to compensate for gyroscope drift. Details about the methods of smartphone accelerometry and its validity for assessing postural control in people with MS have been described previously [22]. MSafe incorporates a React Native interface built with Expo in TypeScript for rapid development and cross-platform capabilities.

In alignment with the Food and Drug Administration guidelines for Digital Health Technology development [23], the design of MSafe was directly informed by a panel of specialists with expertise in various domains of MS care (e.g., researchers, physicians, physical therapists). During one-on-one interviews, these experts identified the evidence-based fall risk factors that are critical to evaluate specifically for the older MS population. For instance, they identified fatigue, fear of falling, balance confidence, objective static and dynamic balance tasks, leg strength, home and outdoor environmental hazards, and vision as important risk factors to assess for older adults with MS. Based on these interviews and findings from the previous literature, we incorporated validated questionnaires and assessments to comprehensively evaluate fall risk for the older MS population (Table 1).

**Figure 2:**
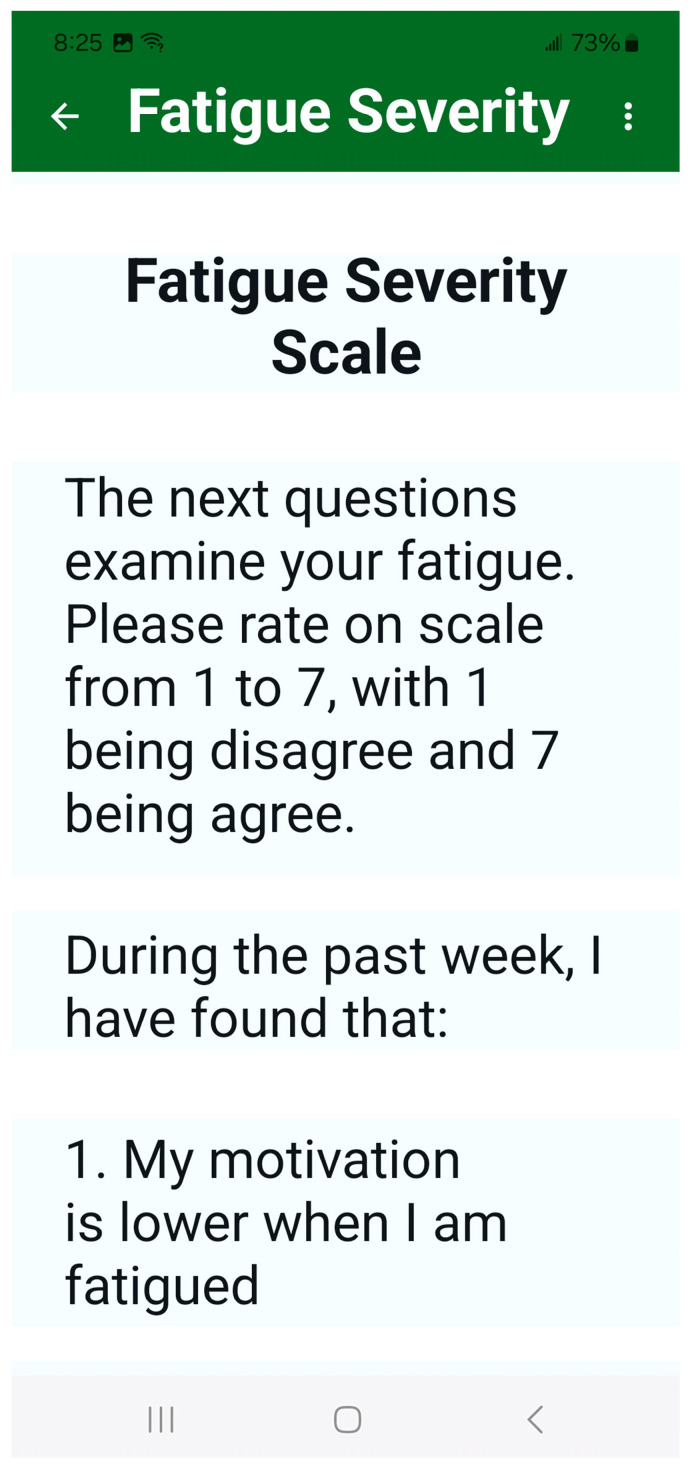
MSafe includes 37 self-report questions. Text describing instructions are in black font against a white background, and headers are in white font against a dark green background.

In addition, the MSafe interface was specifically designed to accommodate the motor, cognitive, and visual impairments that older adults with MS typically experience. Font sizes were a minimum of size 20, with most text at size 28 and titles at size 32. Text describing instructions were in black font against white background, while headers were in white font against dark green background to ensure high contrast (Figure 2). Action buttons and icons were made as large as possible. Each task was presented one at a time to prevent cognitive overload with simple instructions and intuitive navigation.

In total, MSafe guides participants through a series of 37 self-report questions and 9 quantitative cognitive and mobility assessments. Self-reported questions include the following: seven questions of demographics, MS history and type, fall history, and number of medications; six questions of balance confidence using the short form Activities-Specific Balance Confidence (ABC) scale [24]; nine questions of fatigue using the Fatigue Severity Scale (FSS) [25]; eleven questions about the home environment related to paths of travel (four questions), reaching for objects (three questions), and transferring (four questions); and four questions about the outdoor environment most frequently visited by the user. Environmental questions were adapted from the CDC’s Stopping Elderly Accidents, Death, and Injuries checklist [27].

**Figure 3:**
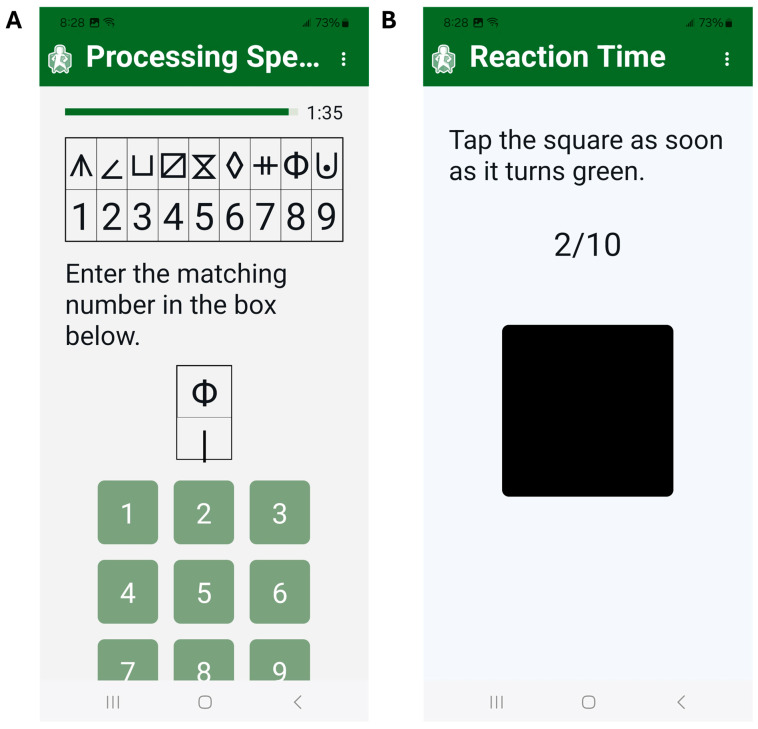
MSafe includes the 130 s symbol–digit matching task to assess processing speed (**A**) and ten trials of the simple reaction time task (**B**).

Assessments include two cognitive tasks and four mobility tasks. The cognitive assessments include a symbol-digit matching task, where users were asked to match as many symbols as possible to the corresponding numbers in 130 s (Figure 3A). The first ten seconds are used as practice, and the number of correct responses in 120 s is recorded. This task was designed to resemble the symbol–digits modality test and measures processing speed [28]. Next is a test of simple reaction time, in which a black square on the screen turns green after a random interval. Users are asked to tap the screen immediately once the square turns green for a total of ten trials (Figure 3B). The time in milliseconds for each trial is recorded, and the average of the ten trials is calculated. Processing speed and reaction time were chosen, as these cognitive domains are associated with falls in older adults and in people with MS [29,30,38].

**Figure 4:**
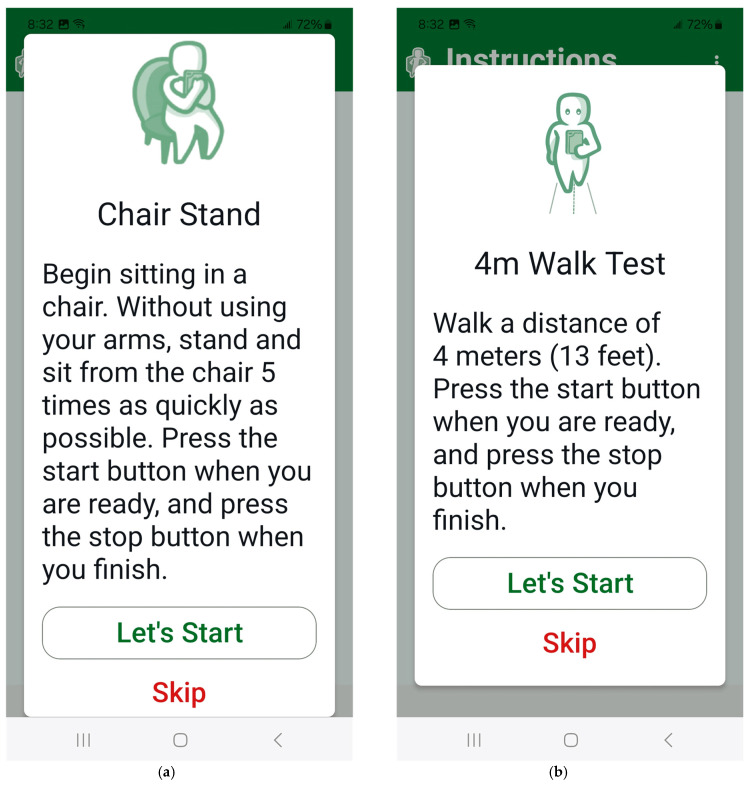
MSafe assesses leg strength with a five-time sit-to-stand (**a**) and gait speed with a 4 m walk test (**b**). Users are asked to tap the screen to start and stop each task.

The mobility tasks include four standing balance tasks, a five-times sit-to-stand, a four-meter walk, and a dual-task four-meter walk. Prior to completing the mobility tasks, users are provided with safety instructions (e.g., stand near a handrail or sturdy surface) and asked to skip any tasks they do not feel safe completing. For the balance tasks, users are instructed to stand for up to 30 s with their feet shoulder-width apart and eyes open, feet shoulder-width apart and eyes closed, feet in tandem position with eyes open, and on a single leg with eyes open. They are instructed to hold the phone against their chest and indicate if they completed the task or if they lost their balance after each task. For the five-time sit-to-stand, users are instructed to tap the screen to start the task and again after completion to measure the total time (Figure 4a) [33]. Lastly, to examine gait speed, users are instructed to complete a single- and dual-task four-meter walk [34]. They tap the screen to begin the walk and again after crossing a four-meter line (Figure 4b). During the dual-task walk, users are instructed to count backwards by 3 from the number 87. For all mobility tasks, tri-axial acceleration is recorded. Whether participants completed or lost their balance during the standing balance tasks is also recorded. The total time in milliseconds for the five-time sit-to-stand, four-meter walk, and four-meter dual-task walk is recorded.

**Figure 5:**
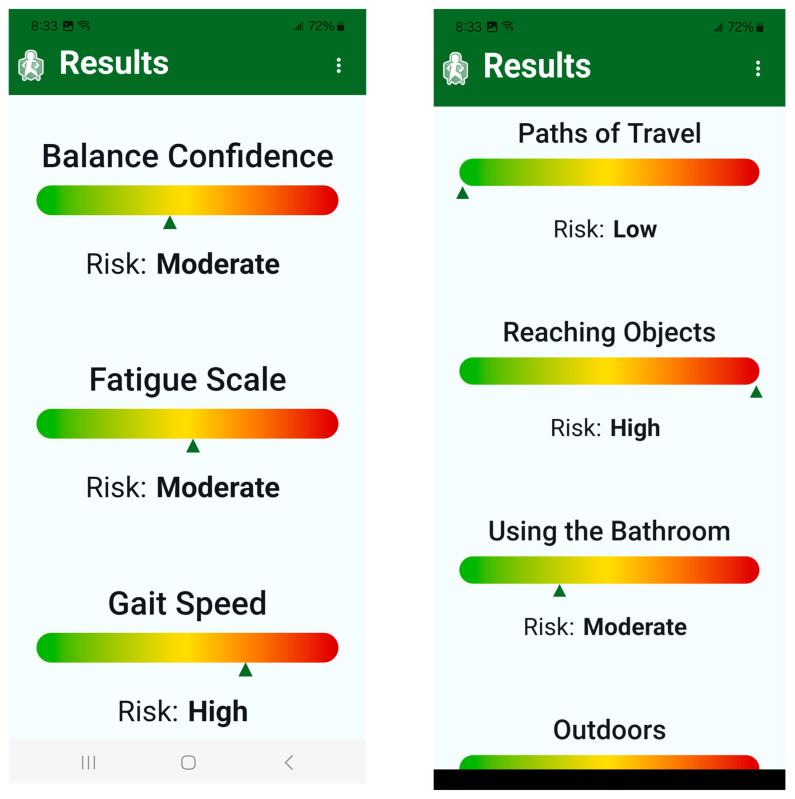
After completing all assessments, MSafe depicts a fall risk report, classifying each domain into low, moderate, or high fall risk on a colored spectrum. Green indicates low risk and red indicates high risk.

After completing the assessments, MSafe presents a fall risk report. This report is grouped by risk factors and presents each risk factor as low, moderate, or high (Figure 5). Each risk factor is weighed equally and separately from each other. Reports were generated for balance confidence, fatigue, processing speed, reaction time, leg strength, balance, and gait, and for each of the environmental components (paths of travel, reaching objects, transferring, outdoors). Established cut-off values were used for each risk factor. For instance, average ABC-6 scores between 85 and 100 were classified as low fall risk, scores between 60 and 84 as moderate fall risk, and scores below 60 as high fall risk [39]. Average FSS scores below four were considered low risk, between four to five as moderate risk, and above five as high risk [40]. Gait speed above 1.1 m/s was considered low risk, between 0.8 and 1.1 m/s as moderate risk, and below 0.8 m/s as high risk [41]. Sit-to-stand time below 12 s was low risk, between 12 and 15 s was moderate risk, and above 15 s was high risk [41]. Symbol digit scores above 40 were low risk, between 30 and 40 as moderate risk, and below 30 as high risk [42]. For environmental hazards, where there are not established cut-offs, questions were worded so that ‘no’ responses indicate a hazard. If over half of the questions in each section (paths of travel, reaching objects, transferring, outdoors) were checked as no, then it was classified as high risk. If half of the responses were checked ‘no’, it was classified as moderate risk, and if less than half of the responses were checked as ‘no’, it was classified as low risk.

### 2.2. MSafe Usability

After designing MSafe and developing the prototype for testing, we conducted a trial wherein 21 older adults with MS used MSafe and then participated in one-on-one semi-structured interviews and questionnaires to assess the app’s usability approved by Georgia State University’s Institutional Review Board. Participants were included if they were 55 years or older, had a physician-confirmed diagnosis of MS, could stand independently for at least 30 s, and could speak and understand English. Participants were excluded if they had any neurological disorder other than MS or if they scored less than 18 on the Montreal Cognitive Assessment [43].

### 2.3. Procedures

After providing written informed consent, participants completed questionnaires related to demographic information and technology experience. They also completed the 16-item ABC scale to assess balance confidence and Falls Efficacy Scale International to examine fear of falling [44,45]. Participants were then presented with a smartphone (Samsung Galaxy A54, Samsung Electronics, Suwon, Republic of Korea) and asked to open MSafe and complete each of the self-report and assessment tasks in the app. Participants were not provided training on how to use the smartphone or MSafe. If participants had questions, they were encouraged to navigate the app as independently as possible, and if they still had questions, a research member would assist. Questions from participants as they used MSafe were recorded by a research assistant.

After completing MSafe, participants participated in the semi-structured interview in which they were asked about their likes, dislikes, desire to use, recommendations for improvement, and potential benefits of MSafe. Semi-structured interviews were video recorded with field notes taken by the research team. Following the interview, participants completed the Systematic Usability Scale (SUS) to examine the overall usability of the app. The SUS is widely used standardized scale consisting of ten questions rated on a five point Likert scale [46]. SUS scores range from 0 to 100, with higher scores representing greater usability. Previous work has indicated that the average technology SUS score is 68, and scores of 80 or above indicate that users are more likely to recommend the device to others [46].

### 2.4. Statistical Analysis

Video recordings were reviewed and transcribed verbatim. Qualitative data from transcripts and field notes were reviewed by two researchers to develop a coding system. Data were assigned codes, and codes with similar content were grouped into thematic categories. SUS ratings were converted using the standard SUS scoring system, and a total SUS score for each participant was calculated on a scale from 0 to 100 as the aggregate of the ten items [47]. Total scores were then averaged across participants.

## 3. Results

### 3.1. Participants

Demographic information of all participants is displayed in Table 2. Participants ranged from 55 to 69 years of age and were majority female (95.2%) with relapse-remitting MS. MS duration ranged from 3 to 40 years. Two-thirds of participants had at least one fall in the past year, and 48% of participants were recurrent fallers. For technology experience, all participants indicated that they frequently use a smartphone. In total, 14% of participants indicated that they do not use a tablet, 29% that they occasionally use a tablet, and 52% that they frequently use a tablet. For vision and hearing impairments, two participants (9.5%) reported serious difficulties with vision even when wearing glasses or contact lenses, and one participant (4.8%) reported serious difficulties with hearing even when wearing hearing aids. Participants spent between 15 and 20 min completing MSafe.

### 3.2. System Usability Scale

The average SUS score was 84.9, with a standard deviation of 14.7. Total scores ranged from 52.5 to 100. A SUS score above 80.3 is considered ‘excellent’ [46]. The question “I think I would use this app frequently” scored the lowest, with 3.1 out of 4. The question, “I thought there was too much inconsistency in the app”, scored the highest: 3.9 out of 4. Three participants provided average SUS scores below 80. Of those participants, two have primary progressive MS, and one was diagnosed with MS 40 years ago, the longest in the sample.

### 3.3. Semi-Structured Interviews

The qualitative analysis of the self-reported desire to use the app, recommendations for improvement, and potential benefits resulted in three themes: (1) simplicity of use, (2) progress monitoring, and (3) guidance and support. Table 3 provides sample quotes for each of these themes.

### 3.4. Simplicity of Use

All participants reported that MSafe was easy to navigate, simple and straightforward, and easy to read and follow. One participant with vision impairments reported that “the questions were easy to read” and “the graphics were very good. [She] could follow them without any problems”. Participants also reported that the “colors were vibrant and great”.

When asked about ways to improve MSafe, four participants suggested having an option for a voice to read instructions out loud. Two of these participants were those who reported having serious difficulty seeing even with glasses or contact lenses. While the instructions were easy to read, these participants liked how other apps they use have this option. In addition, participants suggested improvements to the instructions for the reaction time task. This task instructs participants to touch the screen as soon as a black box turns green. Seven of the participants, however, had trouble understanding the instructions and either pressed the screen when it was black or did not press the screen when it turned green. One participant recommended an animated instruction to demonstrate when to tap the screen.

### 3.5. Progress Monitoring

The second theme was related to tracking and monitoring results. Half of the participants reported that they would like MSafe to track their results to compare their performance from one day to another. One participant, for example, reported that “MS is always changing by the literal minute. If I were to do this first thing in the morning, I’d feel better.” She commented that, since her visit was in the afternoon, she was feeling more tired, and it would be helpful to track her results throughout the day. Another participant reported that she likes to “track everything because [she] knows her cognition and balance changes”. Having “specific numbers and graphs like [her] Apple watch” on MSafe would help improve her tracking.

In addition to using MSafe to monitor one’s own fall risk, four participants also noted that they would like an option to share their results with their physicians. One participant said that she has “had [MS] for 20 years, and it would be nice to show the [changes]” to her neurologist. She added that “it’s hard to explain—over six months, you feel like your walking has changed, but you can’t really identify how. This [app] would tell them how.” Another participant said that she records her symptoms and mood in a journal that she brings to her appointments, and if MSafe had a similar feature, she could share her results with her physician.

Lastly, one participant noted that she would use MSafe because it could help track if she is having a relapse. If she “thinks she’s having a relapse but isn’t quite sure, [she] would use it to track [her symptoms] leading up to it”. She also noted that she would take medications to alleviate her symptoms, but the medications make it more challenging to identify if a relapse is happening. Using a tool like MSafe can help her monitor these changes more objectively.

### 3.6. Alignment and Awareness

The third theme, alignment and awareness, was identified from the fall risk report. When participants received their fall risk report, some participants agreed with the domains that were of higher or lower fall risk. For instance, one participant said she likes “that it gives you an idea of the [fall risk] areas, and I was actually in agreement with most of it”. It reaffirmed areas that she needed to work on in physical therapy. For others, MSafe taught them new areas to improve on. One participant, for instance, “thought [she] had strong legs”, but learned she has weak leg strength after attempting the five-time sit-to-stand test. She wants to now incorporate leg strength exercises into her exercise routine.

## 4. Discussion

The purpose of this study was to develop and evaluate the usability of a multifactorial fall risk app for older adults with MS. Building on our previous prototype [21] and using input from MS experts, we developed MSafe, a fall risk app that leverages built-in sensors and self-reported outcomes to comprehensively evaluate intrinsic and extrinsic fall risk factors for older adults with MS. Guided by previous research and expert feedback, MSafe assesses mobility, cognition, fatigue, balance confidence, vision, and environmental hazards in the home and community. Findings from semi-structured interviews indicated that participants perceived MSafe as simple, easy to use, and valuable for tracking and monitoring fall risk. Participants further reported that the results either confirmed their perceptions of fall risk or increased awareness of their own abilities. The average SUS score was 84.9 ± 14.7, demonstrating excellent usability [48].

Our refined fall risk solution resulted in a user-friendly multifactorial mobile app tailored for older adults with MS. By designing MSafe with large font sizes, contrasting colors, and simple instructions, older adults with MS found the app to be intuitive and user friendly, even among those with visual or cognitive impairments. MSafe may also be useful for older adults with MS in the future to monitor and track their fall risk. Participants noted the variability of their MS and fluctuations in fall risk factors, and they stated that having a tool like MSafe can improve tracking and fall risk management. MSafe also appears to reaffirm participants’ perception of their fall risk or bring awareness to their fall risk. This may help participants adopt new fall prevention strategies or increase adherence to current strategies. Because MSafe took approximately 15–20 min to use, participants can choose to use it on a daily or weekly basis. Interestingly, MSafe’s utility appears to extend beyond tracking fall risk. MSafe may provide potential to track and detect a relapse, and sharing their results with a physician may improve overall healthcare management.

To our knowledge, MSafe is the first mHealth app designed to measure multifactorial fall risk domains and provide tailored results. While numerous mHealth apps exist for the MS population, many focus on managing medications and injections, monitoring specific MS symptoms (e.g., cognition, fatigue), or remote evaluations [16,49]. Other apps, such as Floodlight and MSCopilot, incorporate composite measures of functional domains such as cognition, upper extremity dexterity, and gait function [50,51]. However, these apps were not designed to examine fall risk and do not include other pertinent fall risk factors such as fatigue or environmental hazards. MSafe was developed using a systematic approach, evaluating both intrinsic and extrinsic fall risk factors tailored for the older MS population. By measuring multifactorial fall risk domains, MSafe may also identify those at high fall risk, and, when paired with fall prevention strategies, MSafe can help to reduce their fall risk. For instance, technologies, such as transcranial direct current stimulation, have been used to improve balance impairment and adapted for home use in those with chronic health conditions [52,53]. Pairing the individually tailored results from MSafe with personalized fall prevention strategies offers potential to reduce fall risk and prevent future falls.

The results of this study also inform the next iteration of MSafe, which will include adding a feature to save and view their results after each use. Participants reported that using graphs to depict changes with each use in addition to their raw values will be beneficial to monitor their results over time. Improved instructions for the reaction time task will also be modified, with an audio option to read instructions aloud. To facilitate communication with clinicians, a future iteration will incorporate the ability to save and email results. Our next iteration will also include measures of anxiety and depression, as there is growing evidence that they are associated with future falls in people with MS [54]. When asked for these recommendations, participants often compared MSafe to health apps they currently use, such as fitness trackers (e.g., Fitbit, Garmin) or Apple’s Health app. As these are tools participants are already familiar with, designing a fall risk app to a familiar design may improve usability and adoption. Following these refinements, our next step will be to implement MSafe in home settings and examine how MSafe affects fall risk management behavior. In addition, it is unclear how often older adults with MS should use MSafe to monitor their fall risk. Some risk factors, such as fatigue, fluctuate daily and weekly, while others remain more static [55,56]. Future studies should examine the optimal frequency of monitoring to support personalized fall management strategies.

While this study provides promising initial evidence for the usability of MSafe, it is not without limitations. While we had a diverse sample with a range of education levels, participants had high technology proficiency, especially for smartphone usage. Additionally, because MSafe was tested in a controlled environment, further work is needed to understand its usability and effectiveness in home-based settings. Future studies should examine long-term engagement with MSafe, its impact on fall rates, and whether personalized feedback can lead to behavior change and reduced falls. Incorporating caregiver or clinician feedback may also provide valuable information to enhance the app’s utility. In addition, future studies should aim to include individuals with all types of MS (e.g., primary and secondary progressive, relapse-remitting), as well as those with cerebellar lesions, to examine the usability challenges or differences among these individuals. It is possible that those with primary progressive MS may have a greater accumulation of impairments and faced greater usability challenges as they scored lower on the SUS, but future studies should include larger sample sizes and could evaluate the impact of different impairments (e.g., visual, somatosensory, cognitive) on the usability of MSafe. Lastly, we did not include a measure of depression in MSafe. Despite these limitations, this study also includes many strengths, including incorporating both qualitative and quantitative feedback from participants and designing MSafe using an evidence-based approach.

In conclusion, we took a systematic approach to develop an individualized and comprehensive fall risk app for older adults with MS. Beginning with our initial prototype with Steady-MS, we integrated feedback from clinicals, engineers, and people with MS to develop MSafe. We evaluated the usability of MSafe with older adults with MS using semi-structured interviews and the SUS, with results demonstrating user friendliness, usefulness to monitor fall risk, and increased alignment or awareness of fall risk domains. Our next steps aim to further refine MSafe and implement efficacy testing in home settings to determine the effectiveness of fall risk management.

## Figures and Tables

**Table 1:** MSafe design elements supporting evidence-based multi-domain assessment.

Domain	MSafe Design Element	Validated Construct (Key Refs)
**Balance confidence**	ABC-6 (6 items)	Activities-specific Balance Confidence short form [24]
**Fatigue**	FSS (9 items)	Fatigue Severity Scale [25,26]
**Environmental hazards (home/outdoor)**	CDC STEADI-adapted questions	STEADI ‘Check for Safety’ home safety checklist [27]
**Vision risk**	Self-report prompts	Vision impairment as falls-risk factor [7]
**Cognitive processing speed**	Symbol–Digit matching (120 s; 10 s practice)	Symbol Digit Modalities Test analog [28,29]
**Simple reaction time**	10 trials; mean ms	Reaction/choice stepping RT [30,31]
**Standing balance**	Eyes open/closed feet apart; tandem eyes open; single-leg open (≤30 s) with phone; tri-axial acceleration	mCTSIB and IMU postural sway [22,32]
**Lower-limb strength**	5 Times Sit-to-Stand (user-timed)	5 time sit-to-stand [33]
**Gait speed**	4 m walk (single-task; user-timed)	4 m walk test/4 m gait speed [34,35]
**Dual-task gait cost**	4 m walk with serial 3 s from 87	Dual-task gait cost [36,37]

**Table 2:** Participant characteristics. Continuous variables are reported as mean (standard deviation), and categorial variables are reported as frequency (percentage).

Variable	Participants (n = 21)
Age (years)	61.4 (4.6)
Sex	
Female	20 (95.2%)
Male	1 (4.8%)
Race	
African American or Black	18 (85.7%)
White	3 (14.3)
Education	
High school graduate or equivalent	2 (9.5%)
Vocational training	1 (4.8%)
Some or in-progress college/associate’s degree	5 (23.8%)
Bachelor’s degree	8 (38.1%)
Master’s degree	5 (23.8%)
MS Type	
Relapse Remitting	19 (90.5%)
Primary Progressive	2 (9.5%)
MS Duration (years)	19.8 (8.6)
Walking Aid	9 (42.8%)
Cane	9 (42.8%)
Walker/rollator	4 (19%)
Expanded Disability Status Scale	5.1 (1.0)
Activities Balance Confidence Scale (%)	62.1 (25.4)
Falls Efficacy Scale International	35.9 (12.7)
Montreal Cognitive Assessment	23.1 (2.7)

**Table 3:** Themes identified from interviews, with example quotes from participants.

Theme	Example Quotes
**Simplicity of Use**	“It’s easy to use. It’s user friendly.”
	“It’s pretty much self-explanatory”.
	“It’s very, very easy”.
	“Everything was pretty much clear”.
	“I like how simple it is. You push on that and then the numbers pop up. That’s wonderful”.
**Progress Monitoring**	“MS things are always changing by the literal minute. If I were to do this first thing in the morning, I’d probably be running up or down that hall because I feel better in the morning.”
	“It’s a reinforcement for to see that you started here and you’ve gone there in a month, two months, three months”.
	“I like being able to share the information [*results*] with my doctor. I can share and don’t have to guess.”
	“It’s helpful to explain it [*fall risk*] to me knowing that I could actually track what’s going on, especially the part with the balance score.”
	“It would help to share the information with my doctor. In my case, I’m already concerned about staying upright and not falling, but I think I’ve had this disease for 20 years, and it would be nice to show the difference. It’s hard to explain—over six months, you feel like your walking has changed, but you can’t really identify how. This would tell them how.”
**Alignment and Awareness**	“I like the fact that it kind of gives you an idea of the [fall risk] areas, and I was actually in agreement with most of it.”
	“It reconfirmed some of the kind of things I thought were going on with me”.
	“I think I have strong legs. I really do think I have strong legs, but in this particular situation, I couldn’t do that [*five time sit to stand*].”

## Data Availability

The raw data supporting the conclusions of this article will be made available by the authors on request.
